# The Hospital of St. Francis

**Published:** 1902-07-26

**Authors:** 


### The Institutional Workshop.

### The Hospital of St. Francis.

The dictionary defines a hospital as "a house for the reception and aid of the sick, infirm, or poor," and that being so we must acquit of actual fraudulent intent those who describe as a hospital an institution consisting only of a very small house in which a couple of rooms are used for the reception of patients.

It is well, however, that the facts of the case in regard to the "Hospital of St. Francis" should be known, especially in view of the grandiloquent description of the institution contained in its annual report. "This hospital," we are told, "which is entirely unsectarian, was founded in 1897 for the purposes laid down in its constitution. It is a General Hospital for men, women, and children, and has Special Departments for the treatment of Diseases of the Eye, of Women, of the Throat, Nose and Ear, of the Skin, and of the Teeth."

Here we have an institution for which subscriptions are being collected on the grounds that it is a "General Hospital" with all these "Special Departments," and which displays on the title page of its annual report a list of ten Vice-Presidents, from dukes downwards, of thirteen Lady Patrons, a Council of twenty-one persons, the names of two honorary chaplains, an honorary standing counsel, an honorary junior counsel, an honorary solicitor, an honorary architect, etc., followed by half a page of medical staff, together with the names of an auditor, a secretary, a matron and a dispenser, and yet is but a little house in which the only beds used for patients are crowded into two small rooms about 17 feet by 15 feet, a house which does not possess a bathroom, and in which one water-closet is used indiscriminately for all the in-patients male and female, and for the whole of the staff. The accommodation for patients in this building is put down in the Medical Directory, which we may presume obtained its information from the "hospital" authorities, as being "eight beds and three cots," and we were told by the secretary that the hospital could accommodate ten patients, which comes to nearly the same thing. Reckoning up the cubic space and comparing it with that provided in certain modern hospitals, it would seem that—with their special ventilating appliances which here are absent—the cubic space would be sufficient for perhaps three patients!

Such then is the institution which appeals for subscriptions to the public as a "general hospital" with all sorts of special departments. There could hardly be a stronger argument in favour of people who wish to subscribe money to hospitals and yet are unacquainted with the details of hospital management, sending their subscriptions to some such established fund as the Hospital Sunday Fund or the King Edward's Hospital Fund rather than sending them direct to any so-called hospital whose advertisements excite their sympathies than the impudent assumption of the title "hospital" by this wretched, grubby little house in the New Kent Road. On the occasion of our recent visit we were courteously received by the secretary, to whom our thanks are due for showing us over the building. Let us record our impressions. The houses in the New Kent Road stand a little way back from the street, which has made it possible by building over the forecourt to provide a waiting-room and other offices for the out-patients of this institution. Behind these stands the house, consisting of a basement, two floors, and an attic, and at the back of the house is a very small backyard and a one-storey building, lighted by skylights, which we were told was used as a lumber room. At the back of this is another street, so that the whole establishment stands on a strip of land, which we reckon to be about 18 feet wide, running back from one street to the other, but practically ending where the above-mentioned lumber room begins. At the New Kent Road end of the plot a red brick frontage one storey in height has been erected, on which is the announcement that this is the Hospital of St. Francis. Passing through the entrance door, we find on the right a tiny dispensary, to the left an office, and in front a skylighted room used as a waiting room for out-patients and as the general entrance to the establishment for all purposes. At the farther end of this room we again come to a central passage with a small room partitioned off on each side, the rooms this time being consulting rooms. The fittings of these rooms are meagre, and hardly what might be expected in an institution posing as a "hospital"; but in regard to these matters we do not wish to make any harsh criticism. Good work can be done with very second-rate appliances, if the will is there. It hardly needs saying, however, that better work can be done with better appliances, and that where responsibility is much divided, and the same things are used for many purposes by many men as is the case here, the danger arising from deficient appliances is much increased.

So far we have been in what has evidently at one time been the garden or forecourt. Passing forwards again, and entering the basement of the house itself, we find ourselves in a gloomy and very low room containing a couch, to the left of which is a dark room which we were informed was used for ophthalmoscope work. It contained no lamp, however, but we were told that when used for this purpose, a portable electric lamp was brought in from another room and attached to a switch plug. Passing on again, we found ourselves in the scullery, a very small and low room with a sink and "set pot," and going out at the back door we entered a small back yard in which was a closet for the use in common of the out-patients of both sexes. Returning to the house again, we found that behind the dark room was the staircase, and that opposite the bottom of the stairs was another water closet, this time opening directly inside the house. This closet is the only one which is provided for the whole of the staff, and all the patients, both male and female, as well as for emptying bed-pans, etc.! Going up the stairs we arrived at the male "ward," a room of about 17 feet by 15 feet, the longer dimensions occupying the whole of the front of the house. In this were three beds, and, if we remember right, a cot. The upper part of the windows of this room look out above the roof of the buildings appropriated for out-patients, and the lower portion of the window gets light, and apparently air, from the said out-patients' department, a most objectionable arrangement, and one which ought not to be permitted to continue for a moment in any institution obtaining money from the public on the pretence of being a hospital. The whole of the fittings and the general aspect of this ward were of the most poverty-stricken character, and, indeed, except for the temperature sheets at the head of the beds there was nothing about this room to suggest that it was intended for a hospital ward. Behind this room there was on the left the staircase, such a narrow twisting arrangement as to entirely preclude the proper carriage of a patient seriously ill—and the kitchen, a little room, the extreme measurements of which cannot be more than about 12 feet by 7 feet, and the ceiling of which was within easy reach of the hand. Going up to the next floor we enter the female "ward," if we are to use hospital terms in describing this meagre, grubby, little house. This room is the same size as the one below, but in consequence of the windows not being blocked up and of their getting the fresh air, it is a much more airy and pleasant apartment. It contained three beds, two small cots, and a baby's crib. Above this room, again, is a bedroom for the so-called "probationers," containing three beds. This room is in the roof. At the back, over the kitchen, is a little room occupied by the matron as a bed-sitting-room; above this is a similar room occupied by the senior probationer; and over this, again, is another occupied by the servant. All these back rooms are extremely low, not much, if at all, over 7 feet high; so that while there are three storeys in front there are four behind. As to the appliances for treating the sick, the whole arrangements are beneath contempt. There is a bath which has to be dragged about, but no bathroom; every particle of water has to be carried up to the "wards," and all the slops have to be carried down. The staff engaged in looking after this miserable bit of a place is outrageously large. It consists of one matron, four probationers, one female servant, one porter, one dispenser, one lady clerk to look after the cards of the out-patients, one secretary. Great parade is made of the number of out-patients. That, however, is a detail into which we need not enter, for the out-patients pay for themselves; indeed, so far as "surgery and dispensary expenses" are concerned, they a good deal more than pay for themselves, thus helping to earn money for the in-patient department. The whole thing must stand or fall by its in-patients. We have no means of knowing the average of beds occupied, probably something under half a dozen—a good deal under we should think; but the total expenditure, after deducting the £363 received as payment from out-patients, amounts to £1,068, which, divided among the 158 patients admitted during the year, gives £6 15s. per in-patient, a sum for which any hospital in the neighbourhood would have been delighted to receive them. Thus, even were this institution properly fitted for the work it has taken upon itself, which it is not, this work would be better and more economically done at existing hospitals. There is no place for it, and no excuse for its continued existence.

It may well be asked, then, for whose benefit does this curious excrescence on London charity exist? Certainly it is not for the benefit of the patients. No one can imagine for a moment that the good derived by the patients from being taken into such a house as this is comparable in the slightest degree, if they are really ill, with that which would be gained by their admission to a properly equipped hospital. In the beginning we believe it was the intention of the founders to establish a hospital in which those who were vegetarians might be treated in accordance with their habits, and, if we may so call it, their "faith," and in which the possibility of treating disease on a non-meaty diet might be demonstrated; and if a really well planned and properly conducted hospital were established with this object, although we might not approve, we should at least admit that there was much to be said in its favour. Vegetarianism must, however, be at a miserably low ebb if this is the best show that it can make. We doubt, however, whether this institution, which certainly cannot be properly described as a hospital, can be any more correctly described as vegetarian. Vegetarianism has evidently been thrown over. It is admitted that the medical officers are allowed to order meat, and we may well believe that its present wretched condition is due to the fact that the prospects of the institution have been sacrificed to the ambition of those in power on its council to be able to say that there is at least one hospital in London which definitely excludes vivisection. This is the sort of assertion that is being made, and the veracity of those who make it must be judged by its truth. It is upon the antivivisectionists that we must affix the disgrace of running such an institution as the so-called "Hospital" of St. Francis, and it is to those "antivivisection methods" which have become such a byword that we must attribute the attempt to make the subscribing public believe that the institution which we have just described is a "General Hospital" worthy of the support of the charitable.

